# Natural Exogenous Antioxidant Defense against Changes in Human Skin Fibroblast Proteome Disturbed by UVA Radiation

**DOI:** 10.1155/2020/3216415

**Published:** 2020-11-05

**Authors:** Agnieszka Gęgotek, Pedro Domingues, Elżbieta Skrzydlewska

**Affiliations:** ^1^Department of Analytical Chemistry, Medical University of Bialystok, Mickiewicza 2D, 15-222 Bialystok, Poland; ^2^Mass Spectrometry Center, Department of Chemistry, University of Aveiro, 3810-193 Aveiro, Portugal

## Abstract

Daily exposure of the skin to UVA radiation causes oxidative modifications to cellular components and biomolecules. These include proteins involved in the metabolism and cytoprotection of fibroblasts, and their modification can contribute to the disruption of cell function and the development of skin disorders. Therefore, there remains a need for highly active cytoprotective compounds with antioxidant properties. The purpose of this study was to investigate the effect of ascorbic acid on the activity of rutin against UVA-induced changes in the proteome of human fibroblasts. All analyses were carried out on fibroblasts cultured in a three-dimensional system exposed to UVA radiation and incubated with rutin and ascorbic acid. Their proteomic profile was analyzed using nano-HPLC, which revealed 150 proteins whose expression was significantly altered between treatment conditions. UVA radiation led to changes in the expression of 82 proteins. However, some of these changes were mitigated by rutin and ascorbic acid separately (23 and 25 proteins, respectively) and rutin and ascorbic acid together (23 proteins). UVA radiation has led to the upregulation of proteins involved in gene expression, catalytic processes and antioxidant pathways, and downregulation of proteins with binding activity. Nevertheless, rutin and ascorbic acid used separately or together have countered these changes to varying degrees. Moreover, rutin and ascorbic acid stimulated fibroblasts irradiated by UVA to increase the expression of the signalling molecules responsible for the opening of the transmembrane channels. In the context of the results obtained, the observed cytoprotective effect of the cooperation of rutin and ascorbic acid results not only from the overlapping properties of the compounds. The effect of rutin alone is probably inhibited by its limited bioavailability. Therefore, its interaction with ascorbic acid increases membrane penetration and improves the cytoprotective effect on skin fibroblasts.

## 1. Introduction

**Figure 1:**
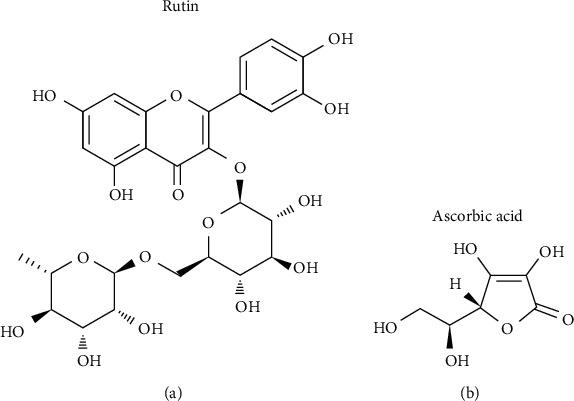
Chemical structure of rutin (a) and ascorbic acid (b).

Rutin is one of the phenolic compounds found in various plant species, where it contributes to the antibacterial properties of the plant [1]. As a glycoside combining the flavonol quercetin and the disaccharide rutinose, it has a chemical structure rich in double bonds and hydroxyl groups (Figure 1(a)) [2]. Rutin also exerts several cytoprotective actions on mammalian cells [3]. Its reported properties include strong antioxidant activities, linked to its ability to directly scavenge free radicals. Rutin can also interact with components of the intracellular antioxidant system, resulting in the restoration of the low-molecular-weight antioxidant pool, increased activity of antioxidant enzymes, and the initiation of expression of cytoprotective genes [4–6]. Studies have also shown that rutin reduces proinflammatory signalling. It does so by reducing the level of reactive oxygen species (ROS) and inhibiting the activity of cyclooxygenase/lipoxygenase, preventing the oxidative metabolism of components of the cell membrane [7, 8].

The cytoprotective properties of rutin contribute to the protection of skin cells exposed to various types of radiation, considerably increasing cell viability [4, 9]. However, its cell-protection properties are limited due to the reduced penetration of the membrane, which increases under the oxidative stress induced by UV radiation [4]. Other factors can also stimulate cells to uptake rutin, including another natural antioxidant, ascorbic acid [10]. Ascorbic acid is a smaller molecule than rutin, and its chemical structure provides a wide antioxidant capacity (Figure 1(b)) [11]. The main intracellular antioxidant function of ascorbic acid is the recycling the lipid-soluble vitamin E by reducing the *α*-tocopheroxyl radicals in the membranes [12]. Ascorbic acid can also protect against inflammation caused by sunburn [13–15]. The synergistic effect between ascorbic acid and rutin has been widely demonstrated in studies analyzing the oral administration of the compounds. These findings were mainly related to their anti-inflammatory and vascular sealing actions [16]. Also, as ascorbic acid is essential for the biosynthesis of collagen [17] and has a direct effect on the absorption of rutin, the potential, synergistic effects of rutin and ascorbic acid in the protection of skin cells have also been described [18–20]. Compounds that exert protective effects on skin cells are of great importance as the cells in different layers of the skin are constantly exposed to harmful environmental factors, including UV radiation, mainly present in sunlight.

UV radiation can disturb various metabolic pathways in skin cells, a feature that has been widely documented in published reports [21, 22]. The main effect of UV radiation is the increased generation of ROS linked to a dysfunction of the antioxidant system [22]. UV radiation is also associated with irreversible oxidative modifications in intracellular molecules, including nucleic acids, phospholipids, and proteins. These modifications can cause metabolic changes, DNA mutations, and even the development of skin cancer [23, 24]. Moreover, ROS, as well as highly reactive lipid peroxidation products, modify the structure of proteins that has been shown for UVB-irradiated fibroblasts [20] and altered protein conformation, leading to a modification of their biological activity [25]. The prevention of this action in skin cells is crucial, mainly in the case of proteins with antioxidant and repairing properties not only in DNA but also in the molecules involved in the transduction of the anti-inflammatory or antiapoptotic signal [25, 26].

While the surface layers of the skin disperse most of the long UV wavelengths, UVA radiation (320–400 nm) shows strong penetration deep into the skin, reaching the dermis, which is mainly composed of fibroblasts [27]. As a result, these fibroblasts have a well-developed cytoprotective system to maintain intracellular homeostasis [28]. However, these natural cellular defense systems are insufficient to cope with the damage associated with UV radiation, requiring the daily use of protective compounds for the skin. Also, the different penetration of these compounds through biological membranes and multilayer cell systems is an additional problem to be solved. So far, the cooperation of rutin and ascorbic acid protects protein structures of UVB-irradiated skin fibroblasts [20]. Therefore, this study was aimed at examining the effect of ascorbic acid on the cytoprotective action of rutin against UVA-induced changes in the proteomic profile of the human skin fibroblasts cultured in a three-dimensional (3D) system.

## 2. Materials and Methods

### 2.1. Cell Culture

**Figure 2:**
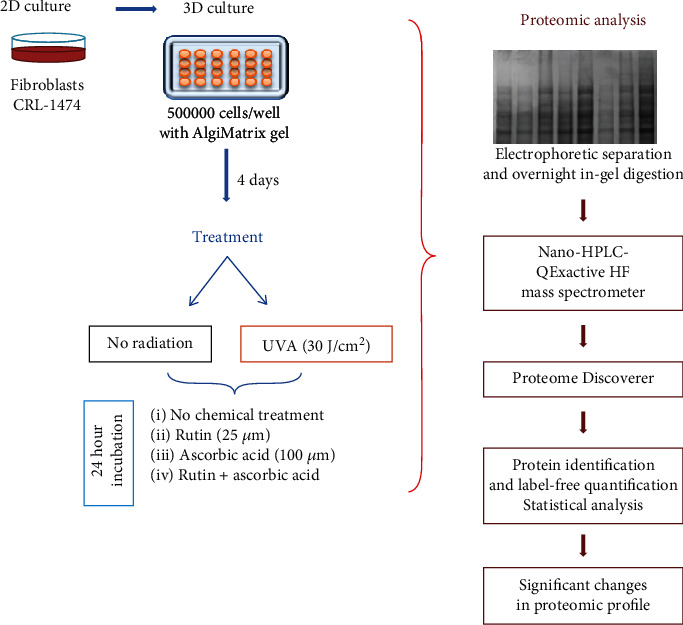
The design of the experiment including cells treatment, sample processing, and statistical data analysis.

The human skin fibroblasts (CRL-1474) were obtained from the American Type Culture Collection (ATCC) and cultured in a humidified atmosphere of 5% CO_2_ at 37°C in a medium composed of Dulbecco's modified Eagle medium (DMEM) with 10% fetal bovine serum (FBS) according to the standard protocol for fibroblasts. To avoid bacterial contamination, medium was supplemented with penicillin (50 U/ml) and streptomycin (50 *μ*g/ml). Following two passages, cells were seeded in 24-well plates (500,000 cells/well) with AlgiMatrix gel (Life Technologies, California, USA) to create a three-dimensional (3D) model. After four days of culturing, the cells were exposed to UVA radiation (Bio-Link Crosslinker BLX 365; Vilber Lourmat, Germany) at a total dose of 20 J/cm^2^ (*λ* = 365 nm). Before irradiation, cells were washed with PBS (4°C). In this buffer, cells in plates put on ice were exposed to the irradiation from 6 lamps with an assembly of 6 W each, which corresponds to 4.2 mW/cm^2^. The distance between cells and lamps was 15 cm. To analyze the effect of rutin and ascorbic acid on UVA-radiated cells, we followed the corresponding methods of Gęgotek et al. [20] that were dedicated changes caused by UVB radiation. Following irradiation, cells were incubated for 24 h in medium containing 25 *μ*M rutin or/and 100 *μ*M ascorbic acid in 0.1% DMSO. Parallelly, control cells were cultured in medium containing 0.1% DMSO. The diagram showing the course of the experiment is shown in Figure 2.

Following incubation, using AlgiMatrix™ dissolving buffer (Life Technologies, California, USA), fibroblasts were collected from the 3D gel. Cells were washed with PBS and lysed by sonification. The total protein content in the lysate was measured using a Bradford assay [29].

### 2.2. Protein Separation and Analysis

Before proteomic analysis, proteins were denatured by mixing with Laemmli buffer that contained 5% 2-mercaptoethanol (1 : 1*v*/*v*) and heated 10 min at 95°C. Preliminary protein separation was prepared on 10% Tris-glycine SDS-PAGE gels. All bands were detected by overnight staining with Coomassie Brilliant Blue R-250. Proteins were sliced and in-gel digested overnight with trypsin (Promega, Madison, WI, USA). Obtained peptide mixture was dissolved in 5% acetonitrile (ACN) + 0.1% formic acid (FA) and separated on 150 mm × 75 mm PepMap RSLC capillary analytical C18 column (Dionex, LC Packings) using Ultimate 3000 HPLC (Dionex, Idstein, Germany). Eluted peptides were analyzed using a QExactive HF mass spectrometer (Thermo Fisher Scientific, Bremen, Germany) operated in a positive mode. Details of the analysis have been described previously [6, 20].

### 2.3. Protein Identification and Label-Free Quantification

Raw data were processed using Proteome Discoverer 2.0 (Thermo Fisher Scientific, Bremen, Germany) and searched against the UniProtKB-SwissProt database (taxonomy: Homo sapiens, release 2019-04). Peptide mass tolerance was set to 10 ppm, MS/MS mass tolerance was set to 0.02 Da, and up to two allowed missed cleavages were used for protein identification. Details of the protein identification have been described previously [6, 20]. Only proteins with at least three identified peptides longer than 6 amino acid residues and at least two unique peptides were selected for further analysis.

### 2.4. Statistical Analysis

Analyses of each sample were performed in three independent experiments. Missing values were estimated using half of the minimum value from the original data imputation. Results from individual protein label-free quantifications were log and *Z*-score transformed. Statistical analysis of data was performed using free available MetaboAnalyst 4.0 software (http://www.metaboanalyst.ca), RStudio software (R version 3.6.2 (2019-12-12)), and Perseus 1.6.10.43. Principal component analysis (PCA) was conducted for exploratory data analysis, with the R package MetaboAnalyst [30]. ANOVA test followed by the Fisher post hoc tests was performed with the R built-in function. Heatmaps were created using the R package pheatmap [31] using “Euclidean” as clustering distance and “ward.D” as the clustering method.

## 3. Results

The proteomic data obtained in this study allowed us to create a list of proteins whose expression was significantly altered in fibroblasts cultured in 3D after irradiation with UVA followed by treatment with rutin and ascorbic acid (Supplementary Table S1). This list includes 150 proteins, 94 of which were identified in control cells and 128 in UVA-irradiated fibroblasts without rutin or ascorbic acid.

**Figure 3:**
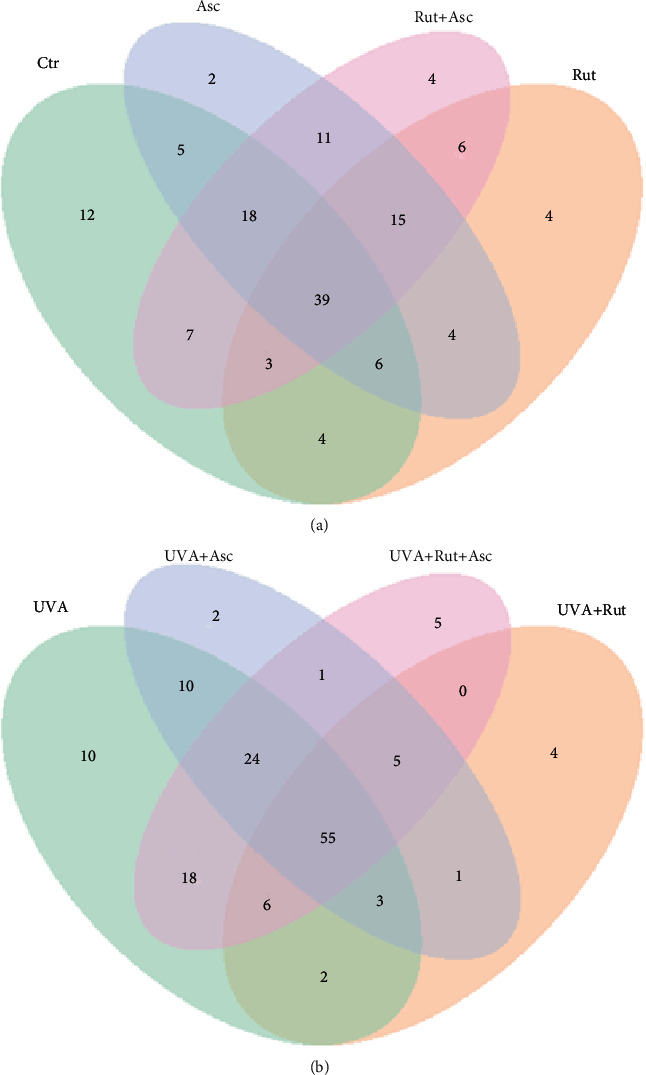
Venn diagram showing the distribution of the number of significantly changed proteins (*p* < 0.05) in the 3D cultured fibroblasts treated with rutin (25 *μ*M) or/and ascorbic acid (100 *μ*M) (a) and following exposure to UVA (20 J/cm^2^) (b). Significant proteins are listed in Supplementary Table 1. Abbreviations: Asc: ascorbic acid; Ctr: control; Rut: rutin.

Treatment with rutin alone induced expression of 4 proteins not found in other treatment groups and 4 other proteins when used with ascorbic acid. The same behaviour was observed in cells treated with rutin/ascorbic acid following UVA irradiation. The number of all significantly altered proteins identified in cells treated with rutin or/and ascorbic acid but without UVA irradiation was generally higher than when cells were irradiated with UVA (46 proteins versus 94). In this case, the chemical treatment induced the expression of 18 proteins which were not found among the 128 proteins expressed in the cells treated only with UVA irradiation (Figure 3).

**Figure 4:**
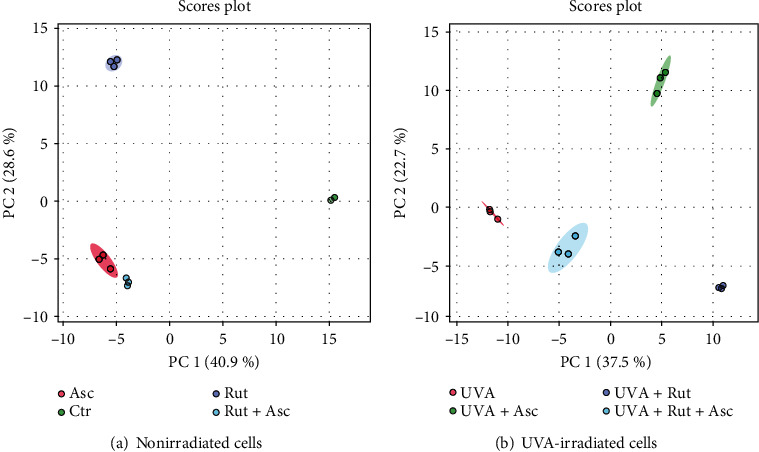
Principal component analysis (PCA) of proteins from the 3D cultured fibroblasts treated with rutin (25 *μ*M) or/and ascorbic acid (100 *μ*M) (a) and following exposure to UVA (20 J/cm^2^) (b). Abbreviations: Asc: ascorbic acid; Ctr: control; Rut: rutin.

Using principal component analysis (PCA), we found that the changes in the proteomic profiles of skin fibroblast cells led to the clustering of the experimental groups (PC1 40.9% and PC2 28.6% for nonirradiated fibroblasts and PC1 37.5% and PC2 22.7% for UVA-irradiated fibroblasts) (Figure 4). In the case of nonirradiated fibroblasts, the control group clustered from the ascorbic acid and the rutin plus ascorbic acid groups, mainly in the lower quadrant. The samples treated with rutin assembled in the upper left quadrant (Figure 4(a)). Observations were different after UVA irradiation, where samples within the treatment groups clustered together mainly in the lower quadrant, and the group treated with ascorbic acid assembled in the upper right quadrant (Figure 4(b)).

**Figure 5:**
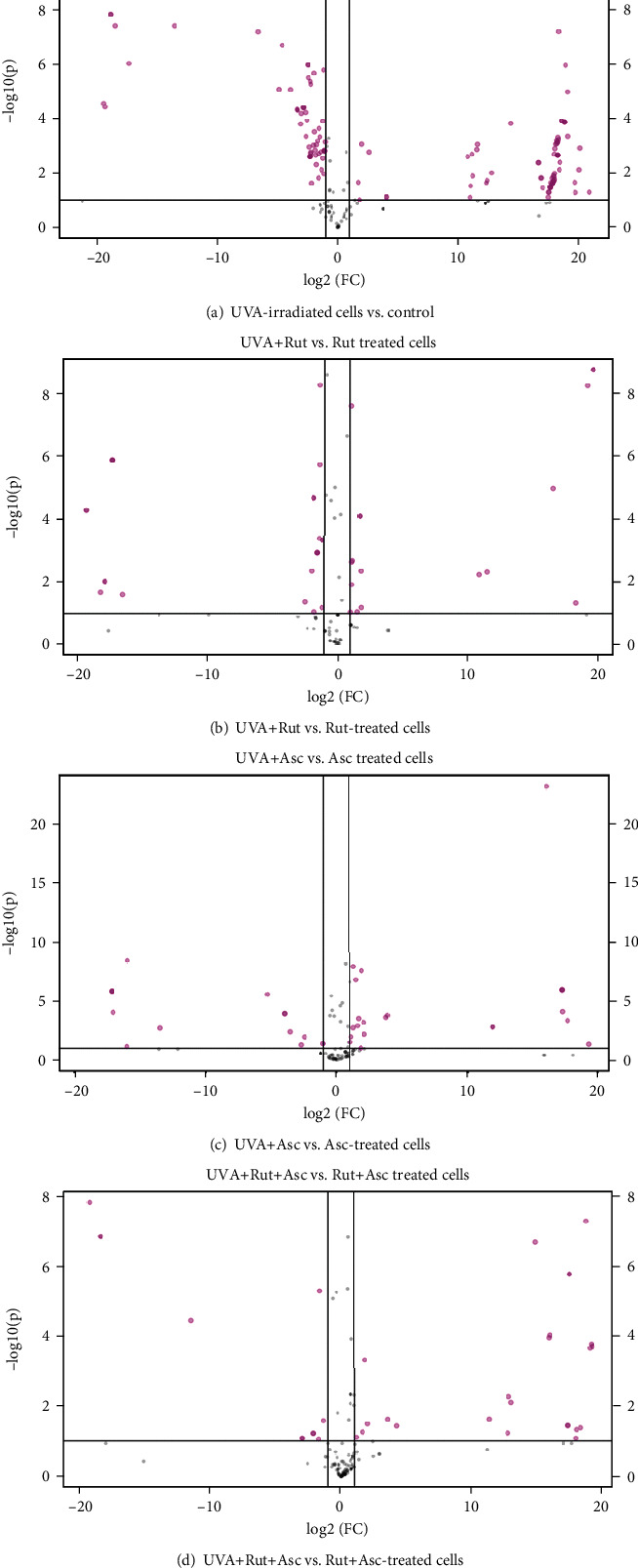
Volcano plots comparing the effect of rutin (25 *μ*M) or/and ascorbic acid (100 *μ*M) on UVA- (20 J/cm^2^) irradiated fibroblasts cultured in a 3D model. Abbreviations: Asc: ascorbic acid; Ctr: control; Rut: rutin. The list of significant proteins, their *p* values, and fold change (FC) are shown in Supplementary Table 2.

**Figure 6:**
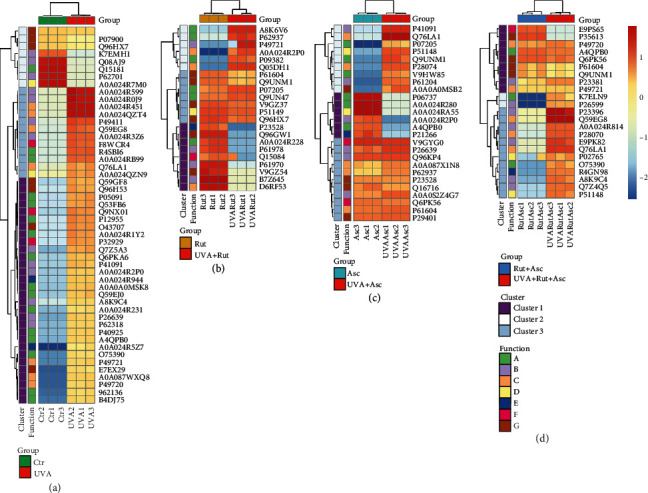
Two-dimensional hierarchical clustering heat map of the significantly different proteins (*p* value < 0.05) from the 3D cultured fibroblasts following exposure to UVA (20 J/cm^2^) (a) and treated separately with rutin (25 *μ*M) (b) or ascorbic acid (100 *μ*M) (c) as well as both compounds together (d). For the creation of heat map A, only the top 50 of significant proteins were used, while all significant proteins were used in heat maps (b–d). Abbreviations: Asc: ascorbic acid; Ctr: control; Rut: rutin. Functions: (A) catalytic activity; (B) transcription/translation regulator; (C) protein degradation regulator; (D) signaling molecule; (E) inflammation regulator; (F) antioxidant activity; (G) binding activity.

The volcano plots comparing the effect of ascorbic acid and rutin on the cells irradiated by UVA also highlight the number of proteins which changed expression under experimental conditions (Figure 5). UVA radiation has caused significant changes in the expression of 82 proteins. However, these changes were attenuated by these cytoprotective compounds to 23 and 25 proteins, respectively, in the case of rutin and ascorbic acid used separately, and 23 proteins in cells treated with rutin plus ascorbic acid. The list of significant proteins and the fold changes (FC) is shown in Supplementary Table S2. The clustering and functions of these proteins can be visualized in the two-dimensional hierarchical clustering heat map (Figure 6). In all the experiments, the clustering of the individual proteins for their similarity in the changes of expression shows that they cluster into three main groups. In the control group, UVA radiation led to upregulation of proteins with mainly catalytic activity or transcription/translation regulatory properties and downregulation of proteins with binding to other molecule activity (Figure 6(a)). Similar observations were made in the case of cells treated with rutin (Figure 6(b)) or with ascorbic acid (Figure 6(c)) before the UVA irradiation. Treatment of cells with these compounds in combination before the UVA irradiation led to upregulation of proteins with antioxidant and transcription/translation activity, as well as regulators of protein degradation. (Figure 6(d)).

## 4. Discussion

Skin cells are exposed to sunlight, which often disrupts cell metabolism due to harmful UVA and UVB radiation [22]. Proteomic analysis of UVB-irradiated fibroblasts indicates that this harmful factor by oxidative stress induction can lead to ROS-dependent changes in protein structures and through enhanced lipid peroxidation products such as reactive aldehydes (4-hydroxynonenal, 4-oxynonenal, and malondialdehyde) cause aldehyde-protein adduct formation [20]. Moreover, UVB has been found as a stimulator of active heat shock protein 90 complex formation that is crucial for proper protein conformation [20]. On the other hand, UVA radiation at the dose used in this study also can modify protein structure, which has been showed as direct oxidation of amino acid residues and advanced glycation end-product creation [32, 33]. These effects involve simultaneous changes at many levels of cell function, including gene transcription, protein biosynthesis, intracellular metabolism, and intercellular signalling [6], without induction of necrosis. Therefore, this study is directly focused on changes in expression of proteins from fibroblasts exposed to UVA radiation in a dose that causes significant changes in cellular metabolism but allows cells to maintain the integrity of nucleus. Moreover, to describe the changes that occur in UVA-irradiated skin cells, it is necessary to understand homeostasis not only at the cell level but also at the tissue level. The use of a three-dimensional cell culture model facilitates the observation of different layers of processes in the skin tissue, including changes in intracellular metabolism and intercellular signalling [34–36].

Fibroblasts are one of the main types of cells that build human skin, but their location in the inner layer of the skin, the dermis, gives them partial protection against external factors by epidermal cells. As a result, fibroblasts are very sensitive to penetrating UVA radiation [37]. To support their self-defense mechanisms, cytoprotective substances (such as rutin) can be administered orally or topically. Many therapeutic properties of rutin as a pharmacological substance have been described [38–40]. However, previous research suggests that regardless of its content in the diet, the low concentrations of rutin in plasma limit its bioavailability to all cells in an organism [41, 42]. However, it has also been shown that rutin can effectively penetrate 3D cultures of skin cells and induce cytoprotective effects even in the deep layers [19]. Moreover, the membrane transport of rutin can be enhanced by ascorbic acid, significantly increasing its action [18].

The results obtained in this study corroborate the previously published data, clearly showing that UVA radiation induces large changes in the proteome of skin fibroblasts [34, 43]. The results also demonstrate that rutin and ascorbic acid exhibit cytoprotective effects, partially preventing UVA-induced changes in the proteome of different cell types [5, 6, 15, 18, 19]. Moreover, recently, it has also been shown that cooperation of rutin and ascorbic acid prevents against UVB induced disturbances in protein structures [20].

The main proteins whose expression was sensitive to UVA radiation were those involved in protein biosynthesis; our data show that UVA radiation significantly upregulates the expression of proteins involved in mRNA transcription processes, including elongation factors (A8K9C4, P26641, and P49411), the translation initiation factor 2 (P41091), ribosomal proteins (A0A024R2P0), and ribonucleoproteins (P62318, Q7Z5A3). This cellular response is aimed at defending against radio-induced stress caused by UVA rays [44].

The cytoprotective action of rutin after UVA irradiation significantly prevents increased expression of translation-inducing proteins, which, in previously published data, has been suggested as a method of inhibiting carcinogenesis [39]. The action of ascorbic acid (separately and in conjunction with rutin) also promotes the expression of these proteins in response to UVA radiation. The changes are very visible not only for the elongation factors and the ribosomal proteins but also in the case of the amino acid-tRNA ligases (tryptophan and threonine-tRNA ligases, P23381 and P26639). These changes in expression lead to the formation of ribosomes, and it is well known that ascorbic acid promotes the increase of the hydroxylation of amino acids in the ribosome. Hydroxylation of amino acids is necessary for the formation of collagen chains, especially under stressful conditions, such as in the case of skin incision wounds [43–45]. Similar changes as those we have described have also been observed in the proteins responsible for the regulation of proteolysis. UVA radiation significantly increases the expression of proteins involved in degradation process, such as the proteasome subunits (A0A087WXQ8, P49720, P49721, and Q59EG8), which is linked to oxidative stress induced by UVA [46]. Rutin counteracts these changes, even causing a reduction in the level of ubiquitin carboxyl-terminal hydrolase (D6RF53) in cells, following exposure to UVA. The reduction of this protein limits the generation of ubiquitin monomers from protein adducts [47], thereby preventing labelling of proteins for degradation. Similar effects of proteasome inhibition by flavonoids have already been observed [48].

In our study, we have found that ascorbic acid also modestly reduced the expression of several proteins involved in degradation following UVA radiation. However, some of these proteins were more strongly upregulated than in fibroblasts without treatment with ascorbic acid. A similar complex and ambiguous effect of ascorbic acid on proteasomal activity has also been observed, mainly in cells with disturbed metabolism, such as cancer cells [49, 50]. The response of fibroblasts irradiated with UVA to the actions of rutin and ascorbic acid shows that these compounds, when administered together, significantly prevent the degradation of proteins in irradiated cells. This observation may be related to structural changes in proteins caused by UVA radiation. These modifications could make the proteins dysfunctional and be eliminated by degradation, as previously observed in keratinocytes or UVB-irradiated fibroblasts [20, 51]. However, the cytoprotective effect of rutin with ascorbic acid protects proteins from oxidative damage, and high levels of proteasomal subunits in the cytoplasm are not necessary.

Other groups of proteins highly upregulated by UVA radiation in 3D cultured skin fibroblasts were proteins with catalytic activity, in particular in glycolytic processes. Similar changes have been previously described also for UVB-irradiated fibroblasts [20]. Reducing stress induced by exposure to UV requires a lot of energy from the cells, which induces an additional consumption of glucose and is associated with accelerated cellular metabolism [52]. Rutin significantly limits the number of catalytic proteins participating in energy reactions following UVA radiation and has already been shown to impair energy processes in the mitochondria of stressed cells by inhibiting substrate oxidation, as well as acting as an uncoupler of oxidative phosphorylation [53]. Ascorbic acid also lowers the levels of proteins involved in glycogenolysis (glycogen phosphorylase, P06737) and interfering with glutaminolysis (phosphoserine aminotransferase, A0A024R280). The effect of ascorbic acid on ATP generation and energy metabolism is closely related to its concentration, as well as to the conditions in which the cells are found [54]. As has been shown for UVB-irradiated fibroblasts and other skin cells (keratinocytes), ascorbic acid, as well as rutin, also supports the production of aerobic energy by cells, especially under stress [19, 20]. However, in fibroblasts, these two processes overlap when cells were treated with rutin and ascorbic acid. This mechanism provides cells with a reduced intensity of electron flow in the mitochondrial respiratory chain and thus reduces the likelihood of generation of free radicals [55].

The antioxidant action of rutin and ascorbic acid, which according to the literature is mainly based on their free radical scavenging activity [56], leads to a reduced expression of the proteins of the antioxidant system which are strongly activated by UVA radiation. An example of such an action is the effect on the thioredoxin system, previously observed also in the case of UVB-irradiated cells [20]. UVA radiation significantly changes the level and activity of proteins in this system [19, 57], including the thioredoxin-like protein 4B (Q9NX01), as found in this study. The antioxidant activity of this system is based on the reduction of NADPH-dependent protein disulfide. This is ensured by the structure of thioredoxin, which, as a small thiol-active polypeptide, is an essential electron donor for UV-oxidized proteins [58]. UVA radiation also induces the expression of cystathionine gamma-lyase (CTH, P32929), which catalyzes the biosynthesis of cysteine or thiocysteine. The increased activity of CTH is the response of cells to the increased demand for thiol groups under oxidative stress [59], an effect observed following UVA irradiation. Due to their effective antioxidant properties, rutin and ascorbic acid probably reduce free radicals so vigorously that they prevent the reduction of the thiol group pool, which occurs after UVA irradiation of fibroblasts.

A slightly different situation has been observed in keratinocytes cultured in a 3D model. In these cells, treatment with antioxidant compounds additionally supports the natural cellular antioxidant system [19]. Moreover, when used separately, rutin and ascorbic acid reduce the expression of other antioxidant proteins in skin fibroblasts after UVA irradiation, altering the level of reduced thiol groups such as glutathione S-transferase (P21266) and the protein disulfide-isomerase (Q15084). However, rutin and ascorbic acid used together not only prevent UVA-induced activation of the antioxidant system but also silence the machinery necessary for protein repair, observed by the decrease of the heat shock cognate protein (E9PS65). Similar effects of flavonoids on the level of heat shock proteins have already been observed in cancer cells, a mechanism that was important for removing mutated cells from the organism [60].

The additional cytoprotective mechanism of skin fibroblasts against UVA radiation is their ability to provide multilayer protection for skin cells [61]. In such a system, communication between cells by transmission signal factors is highly developed [62]. However, as previously shown, UV radiation also disturbs the functioning of the skin cell at this level [19, 63]. In the case of the epidermis, UV radiation substantially induces proinflammatory and proapoptotic signals, while rutin and ascorbic acid prevent these changes [19]. In this study focused on dermal fibroblasts, the main proteins with modified expression (from the top 50 significant proteins) after UVA irradiation are mainly associated with the functioning of intracellular membranes, including voltage-dependent anion channel 2 (VDAC, A0A024QZN9) and transmembrane emp24 domain-containing protein 9 (TMED, A0A024R7M0). VDAC is a major protein in the outer mitochondrial membrane (and, to a lesser extent, the cell membrane). It facilitates the flow of ions and small hydrophilic molecules through the membrane [64]. Ascorbic acid is an example of such a molecule, but, conversely, it is a potent inhibitor of other voltage-dependent channels in the mitochondrial membrane [65]. Moreover, rutin has also been shown to act as a VDAC inhibitor [66]. Therefore, in fibroblasts cultured in 3D, we observed a UV-induced increase in the expression of VDAC and the complete elimination of this effect following treatment of the cells with rutin and ascorbic acid. However, the role of TMED following UVA irradiation is still unclear; the downregulation of this protein by rutin and ascorbic acid may prevent metabolic dysfunctions [67].

Rutin, in combination with ascorbic acid, also causes UV-irradiated fibroblasts to upregulate the expression of Ras-related protein Rab-5C (P51148). This protein activity is also closely linked to the membrane-bound proteins and GTP hydrolysis, which participates in the regulation of the secretion of extracellular signalling molecules [68]. Other polyphenols (e.g., resveratrol) can upregulate Ras-related protein, Rab [69, 70]. However, rutin has not been shown to have these properties. This study shows that it is only possible in association with ascorbic acid. Ascorbic acid promotes intercellular communication under oxidative stress and protects cells from the harmful effects of UVA radiation.

## 5. Conclusions

In conclusion, the strong antioxidant properties of rutin, as well as its effects on intracellular and intercellular signalling, are probably inhibited by its limited bioavailability. In the context of the results obtained in this study, this natural polyphenol requires to be administered with ascorbic acid not only to increase its membrane penetration but also to complement its cytoprotective effect on skin cells irradiated with UV. Analysis of the mechanisms of action of rutin and ascorbic acid in different types of skin cells, such as fibroblasts and keratinocytes [19], draws particular attention to the changes in the proteome. These changes mainly involved proteins associated with gene expression, signal transduction, and the antioxidant response. Thus, the coadministration of rutin and ascorbic acid can protect against the harmful effects induced by UVA rays and these findings can be useful for the prevention of skin diseases linked to irradiation and oxidation processes.

## Data Availability

The mass spectrometry proteomics data have been deposited to the ProteomeXchange Consortium via the PRIDE [70] partner repository with the dataset identifier PXD018730.

## Supplementary Materials

Supplementary materials Supplementary Table 1: list of significant proteins indicated in the 3D cultured fibroblasts treated with rutin (25 *μ*M) or/and ascorbic acid (100 *μ*M) and following exposure to UVA (20 J/cm^2^). Supplementary Table 2: list of significant proteins form volcano plots (Figure 5) comparing the effect of rutin (25 *μ*M) or/and ascorbic acid (100 *μ*M) on UVA- (20 J/cm^2^) irradiated fibroblasts cultured in a 3D model.
